# Paradoxical Hyperkalemia in Bartter Syndrome: A Case Report of Severe Resistant Hyperkalemia Requiring Hemodialysis

**DOI:** 10.1155/crin/2644098

**Published:** 2025-12-11

**Authors:** Abhishek Vadher, Mehwish Zeb, Shayne Joseph, Sujata Kambhatla, Chadi Saad

**Affiliations:** ^1^ Department of Internal Medicine, Garden City Hospital, Michigan State University, Garden City, Michigan, USA, msu.edu; ^2^ Department of Nephrology, Garden City Hospital, Michigan State University, Garden City, Michigan, USA, msu.edu

## Abstract

(https://ce.mayo.edu/content/abstract-submission-mayo-clinic-nephrology-hypertension-and-kidney-transplantation-update-2). Bartter syndrome is characterized by severe hypokalemia due to renal tubular defects. A female in late 30s with Bartter syndrome Type IV presented with dizziness. She had been managing severe hypokalemia with intravenous potassium supplementation via an implanted port. She missed her scheduled potassium infusion and presented to the emergency department with a critically high potassium level of 8.2 mmol/L. Additionally, she was diagnosed with acute kidney injury, with a GFR of 39 mL/min down from 72 at her baseline. Her EKG showed QRS widening and tall T waves. Despite administration of multiple shifters, her potassium levels remained elevated. Urgent hemodialysis was initiated, temporarily reducing her potassium levels, but with a rebound to 9.1 mmol/L within hours. After three cycles of hemodialysis over 36 h, her potassium levels finally normalized to 4.7 mmol/L, and no further dialysis was required.

## 1. Introduction

Bartter syndrome is autosomal recessive disorder of salt reabsorption which results in extracellular fluid volume depletion and a normal or low blood pressure [1]. There are several electrolyte abnormalities, low potassium and low chloride being most common and, in some cases, hypomagnesemia. High renin, secondary hyperaldosteronism, along with elevated levels of prostaglandin E2 are also seen. Metabolic alkalosis is also commonly witnessed. The most common presentation is in infancy with failure to thrive. According to the site of the impaired salt transport, various phenotypes are classified. The clinically significant variants are antenatal/neonatal Bartter syndrome, classic Bartter syndrome, and Gitelman syndrome.

This case underscores the rarity and resistant nature of paradoxical hyperkalemia in Bartter syndrome, a condition typically associated with severe hypokalemia. Our case highlights the need for clinicians to remain vigilant for hyperkalemia in Bartter syndrome, which may require unique management strategies. Further research is necessary to investigate genetic variants and individual patient factors.

## 2. Case Report

A 39‐year‐old female with Bartter syndrome Type IV diagnosed at the age of 2 years, fibromyalgia, and insomnia presented with dizziness. She had been managing severe hypokalemia with intravenous potassium supplementation via an implanted port since last 7‐8 years. Her home medication regimen was potassium chloride solution po 100 mEQ three times a day and potassium infusions 80–120 mEQ every Monday, Wednesday, and Friday based on her potassium levels which was started because she could not tolerate higher doses of po KCl due to esophagitis. Her other home medications included aldactone 100 mg and amiloride 5 mg daily.

The patient reported missing her last scheduled potassium infusion due to multiple episodes of vomiting and multiple episodes of diarrhea. On presentation, laboratory results revealed a critically elevated serum potassium level of 8.2 mmol/L and evidence of acute kidney injury (AKI), with an estimated glomerular filtration rate (eGFR) of 39 mL/min/1.73 m^2^, decreased from her baseline of 72 mL/min/1.73 m^2^.

*Other laboratory findings* were as follows:

• Arterial blood gas: pH 7.351, pCO_2_ 40.1 mmHg, pO_2_ 90 mmHg, HCO_3_^−^ 21.7 mmol/L
• Serum sodium: 122 mmol/L
• Magnesium: 2.01 mg/dL
• Glucose: 84 mg/dL
• Chloride: 98 mmol/L
• Liver function tests: within normal limits
• BUN: 17 mg/dL
• Serum creatinine: 1.69 mg/dL (baseline 1.12–1.23 mg/dL)

**Figure 1:**
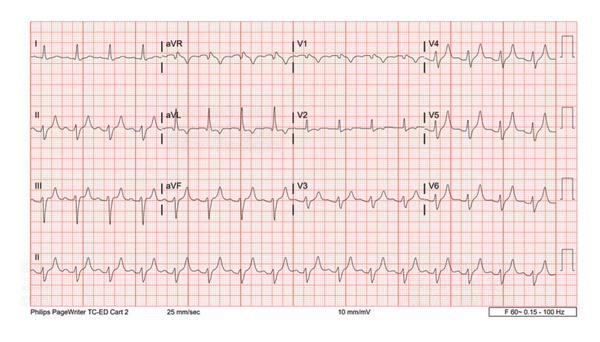
EKG on arrival.

EKG on arrival in ED showed QRS widening to 138, tall T waves, and PR elevated to 195 (Figure 1).

**Figure 2:**
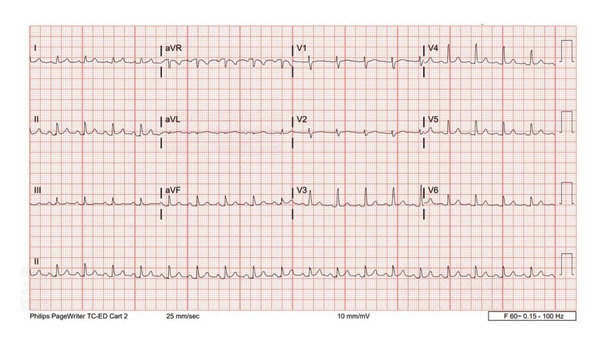
Repeat EKG showed resolution of tall T waves, PR, and QTc normalizes.

Initial management included administration of 1 gm calcium chloride. Shifters were administered with insulin 10 IU and 25 gm D50% and Lokelma 15 mg administered for potassium elimination. Repeat EKG showed no changes of hyperkalemia (Figure 2). Repeat serum potassium was 7.7 mmol/L prompting additional shifters with insulin and dextrose which reduced potassium to 6.6 mmol/L. She was transferred to Intensive Care Unit (ICU) for closer monitoring.

Repeat serum potassium after 6 h was 9.1 mmol/L with EKG findings of tall T waves, PR, and QT prolongation. Due to refractory hyperkalemia, hemodialysis was initiated for potassium elimination from the body. She underwent 2 h of hemodialysis with following parameters.

*First HD session*:

• Duration: 2 h
• Dialyzate composition: K^+^ 1 mEq/L, Ca^2+^ 2.5 mEq/L, bicarbonate 35 mEq/L, Na^+^ 138 mEq/L
• Dialyzer: F160
• Blood flow: 250 mL/min
• Dialyzate flow: 500 mL/min

Post dialysis serum potassium at 3 h was 7.0 mmol/L. Repeat serum potassium after 6 h was 8.7 mmol/L. Over the next 9 h, she received a total of *5 gm IV calcium gluconate, 100 mEq sodium bicarbonate* (followed by a continuous bicarbonate infusion), and an additional *10 gm Lokelma*.

*Second HD session* was performed 12–18 h after the first, under similar parameters (2 h, K^+^ 1 mEq/L bath, Ca^2+^ 2.5 mEq/L, bicarbonate 35 mEq/L, Na^+^ 138 mEq/L, blood flow 350 mL/min, and dialyzate flow 600 mL/min). Postdialysis potassium was *6.0 mmol/L* but again rose to *9.1 mmol/L* after 6 h.

*Third HD session* was conducted approximately 9 h later (2 h, K^+^ 1 mEq/L bath, Ca^2+^ 2.5 mEq/L, bicarbonate 35 mEq/L, Na^+^ 138 mEq/L, blood flow 350 mL/min, and dialyzate flow 800 mL/min), resulting in a postdialysis potassium of *4.7 mmol/L*. No further hemodialysis sessions were required thereafter. Her potassium was checked every 12 hourly for the next 48 h, and it stayed eukalemic.

## 3. Discussion

Bartter syndrome is a rare genetic disorder identified by Bartter et al. in 1962, characterized by a salt‐wasting tubulopathy. This condition leads to hyperplasia of the juxtaglomerular apparatus, resulting in hyperreninemia, adrenal cortical hyperplasia with normotensive hyperaldosteronism, and hypokalemic metabolic alkalosis [2]. As an autosomal recessive disorder, its incidence is estimated at approximately 1 in 1,000,000 individuals [3]. The syndrome is caused by loss‐of‐function mutations in ion channels located in the thick ascending limb (TAL) of the loop of Henle, which impairs renal salt reabsorption and precipitates significant hypokalemia, hypochloremia, and hypercalciuria [3]. In the patient in this case report, we believe multiple episodes of vomiting and diarrhea prior to admission caused hypovolemia and prerenal AKI. K was 6.0 on 2 days prior to admission and potassium infusion was held on 2 days prior to hospital admission. This is also evident by low sodium of 122 in the ER which is consistent with hypovolemic hypovolemia. We suspect that hypovolemia triggered AKI and then DCT and collecting ducts started retaining potassium.

### 3.1. Clinical Features and Diagnosis

Hypokalemia and hypochloremia are hallmarks of Bartter syndrome. Clinical evaluation typically involves urine electrolyte analysis, which reveals elevated levels of sodium, potassium, chloride, calcium, and prostaglandin E2 [4]. Notably, 24‐h urinary calcium excretion is elevated, distinguishing Bartter syndrome from Gitelman syndrome, which is characterized by low calcium excretion [4]. Blood analyses usually indicate elevated renin and aldosterone levels, and arterial blood gas tests commonly show metabolic alkalosis [4]. Diagnosis can be confirmed through genetic testing using next‐generation sequencing targeting relevant genes such as SLC12A1, KCNJ1, CLCNKB, CLCNKA, BSND, and MAGED2 [4].

Bartter syndrome arises from various genetic mutations that disrupt salt reabsorption in the TAL of the loop of Henle [5–8]. These mutations affect different ion channels and transporters, resulting in multiple subtypes:

#### 3.1.1. Type 1

Mutations in the SLC12A1 gene impair the function of the NKCC2 transporter, leading to salt wasting, extracellular volume contraction, and elevated renin, Angiotensin II, aldosterone, and Prostaglandin E2 levels. This results in hypokalemia and metabolic alkalosis due to increased distal sodium delivery and potassium excretion [5–8].

#### 3.1.2. Type 2

Mutations in the KCNJ1 gene affect the ROMK channel, essential for recycling potassium in the TAL. This leads to reduced sodium reabsorption by NKCC2 and similar electrolyte disturbances as in Type 1 Bartter syndrome. ROMK dysfunction in the late DCT, and collecting duct can cause transient hyperkalemic metabolic acidosis [5–8].

#### 3.1.3. Type 3

Mutations in the CLCNKB gene cause the loss of function of the ClC‐Kb chloride channel, impairing chloride reabsorption in the TAL and causing NKCC2 dysfunction. This results in increased urine volume with high sodium and chloride content reaching the distal nephron. Increased distal sodium reabsorption leads to potassium wasting, hypokalemia, and metabolic alkalosis through mechanisms similar to Type 1. Higher expression of ClC‐Kb in the DCT compared to the TAL results in partial loss of NCC function, leading to hypocalciuria and hypomagnesemia resembling Gitelman syndrome [5–8].

#### 3.1.4. Type 4

Mutations in the BSND gene result in the loss of function of Barttin, a necessary accessory protein for ClC‐Ka and ClC‐Kb chloride channels. This inactivation abolishes chloride transport in the TAL and DCT, leading to severe salt wasting and reduced concentrating ability. Hypomagnesemia and sensorineural deafness occur due to chloride transport defects in the inner ear and nephron [5–8].

#### 3.1.5. Type 5

Mutations in the MAGED2 gene disrupt the MAGE‐D2 protein, affecting the expression and activity of NKCC2 and the thiazide‐sensitive sodium chloride cotransporter (NCCT), reducing their function on cell surfaces. This results in reduced salt reabsorption and associated electrolyte imbalances [5–8].

Bartter syndrome presents with a range of clinical features, primarily stemming from renal salt wasting and electrolyte imbalances [5–7, 9]. Patients often exhibit polyuria and hyposthenuria, leading to signs of hypovolemia and dehydration such as normal or low blood pressure, dizziness, fatigue, and polydipsia [5–7, 9]. Early life symptoms include poor feeding, rapid weight loss after birth, and failure to thrive or severe growth delay. Recurrent vomiting, diarrhea, and fever are also common [5–7, 9]. Hypokalemia‐related symptoms such as constipation, muscle cramps, weakness, hypochloremic and hypokalemic metabolic alkalosis, and nephrocalcinosis are frequently observed [5–7, 9]. Tetany due to hypomagnesemia, along with distinctive facial features, such as a triangular face, prominent forehead, large eyes, protruding ears, and a drooping mouth, are noted [5–7, 9]. Intellectual disability, strabismus, convulsions, and increased susceptibility to infections may also be present. In more severe cases, patients might experience chronic hypokalemia‐related complications such as prolonged QT interval, cardiac arrhythmias, rhabdomyolysis, syncope, and paralysis [7, 9].

Hypokalemia is defined as a serum potassium level below 3.5 mEq/L (3.5 mmol/L) and can be categorized into disorders of internal balance, involving potassium movement between intracellular and extracellular compartments, and disorders of external balance, concerning potassium intake and output. Typically, serum potassium levels above 3.0 mEq/L (3.0 mmol/L) do not present symptoms. Given that the ratio of intracellular to extracellular potassium is crucial for the membrane potential of electrically active tissues, hypokalemia can lead to muscle weakness or paralysis, decreased gastrointestinal motility or ileus, and cardiac arrhythmias. Electrocardiogram (ECG) changes associated with hypokalemia include ST‐segment depression, reduced T‐wave amplitude, and increased U‐wave amplitude. In Types 1, 3, and 5 Bartter syndrome, hypokalemia is a common feature, whereas transient hyperkalemia (serum potassium above 5.5 mmol/L) is observed in Type 2 Bartter syndrome during the first few days of life [5–7, 9].

In this case report of an adult patient with Bartter syndrome presenting with hyperkalemia, we document a rare and atypical manifestation of a condition typically characterized by hypokalemia. While Bartter syndrome is well‐known for causing hypokalemic metabolic alkalosis due to impaired renal handling of potassium, the occurrence of hyperkalemia in adult patients is highly unusual. This presentation necessitates a careful reevaluation of electrolyte disturbances in such patients, especially considering the well‐documented mutations affecting renal tubular function. The pathophysiological mechanisms leading to hyperkalemia in Bartter syndrome may involve transient disruptions in potassium homeostasis, as has been reported in neonates with Type 2 Bartter syndrome [10, 11]. However, its manifestation in adulthood, as observed in this case, remains largely unexplored in the literature. This case underscores the importance of considering variable electrolyte presentations in Bartter syndrome and highlights the need for individualized treatment approaches in managing such atypical cases.

### 3.2. Understanding Hyperkalemia

Hyperkalemia, defined as a serum potassium level exceeding 5.0–5.5 mEq/L, poses significant risks when levels rise, particularly causing cardiac arrhythmias, muscle weakness, or paralysis [12]. Though mild elevations are often asymptomatic, dangerous symptoms typically emerge with potassium levels above 6.5 mEq/L. Mild to moderate hyperkalemia often presents with nonspecific symptoms such as generalized weakness, fatigue, nausea, vomiting, abdominal cramping, and diarrhea [13]. However, severe hyperkalemia can result in critical conditions such as cardiac arrhythmias and muscle paralysis [13]. Potassium and sodium are crucial for proper myocardial function, and their concentration gradients are tightly regulated. These gradients are maintained by the sodium‐potassium ATPase pumps on the cell membrane, which transport sodium out of the cell and potassium into it. An increase in extracellular potassium reduces this concentration gradient and lowers the resting membrane potential [13]. This alteration affects the activation of sodium channels, leading to reduced inward sodium current, prolonged impulse conduction, and delayed depolarization. The myocardium, highly sensitive to potassium fluctuations, exhibits a range of ECG changes in response to hyperkalemia [13].

Hyperkalemia can result from increased potassium intake, especially in patients with renal impairment, extracellular shifts (as seen with rhabdomyolysis or metabolic acidosis), or impaired potassium excretion, particularly in kidney disease [12, 13]. In a patient with hyperkalemia, the first test to order is an ECG, as the most dangerous complication of hyperkalemia is its impact on cardiac function, which can lead to dysrhythmias and potentially fatal outcomes. Hyperkalemia induces characteristic ECG changes in a dose‐dependent manner [12]. Specifically,

• When serum potassium levels range from 5.5 to 6.5 mEq/L, the ECG typically shows tall, peaked T waves [12].
• At levels between 6.5 and 7.5 mEq/L, there is often a loss of P waves [12].
• When potassium levels reach 7.0 to 8.0 mEq/L, the QRS complex begins to widen [12].
• At levels from 8.0 to 10.0 mEq/L, patients may exhibit severe arrhythmias, a sine wave pattern, and potentially asystole [12].

It is crucial to recognize that the rate of increase in serum potassium is more significant than the absolute level in determining ECG changes. Chronic hyperkalemia may not always produce notable ECG alterations even at elevated levels, while sudden spikes in potassium can cause significant ECG changes at much lower concentrations. Notable ECG features of hyperkalemia include small or absent P waves, prolonged PR interval, augmented R waves, wide QRS complexes, and peaked T waves [12, 13].

Immediate management includes discontinuation of potassium sources, stabilizing cardiac membranes with calcium, and promoting intracellular potassium shifts through insulin, glucose, or beta‐agonists. Hemodialysis is the preferred treatment for life‐threatening hyperkalemia, particularly in patients with impaired renal function, severe rhabdomyolysis, or hyperkalemia unresponsive to other medical interventions. During hemodialysis, plasma potassium levels decrease by approximately 1 mmol/L in the first hour, with a total reduction of about 2 mmol/L after 3 h. The potassium level then stabilizes, reaching a plateau at around 4 h [14].

## 4. Conclusion

In conclusion, while hyperkalemia is not a classic feature of Bartter syndrome, its potential occurrence underscores the importance of comprehensive monitoring and individualized management strategies for affected patients. A thorough understanding of the complex interplay among renal function, medication effects, and dietary factors is essential for preventing and effectively managing hyperkalemia within this unique patient population. By integrating a multidisciplinary approach, healthcare providers can significantly improve clinical outcomes and quality of life for individuals living with Bartter syndrome.

## Ethics Statement

No written consent was obtained from the patient for publication of this case report. All procedures followed were in accordance with the ethical standards.

## Consent

Please see the Ethics Statement.

## Disclosure

All authors reviewed and approved the final version of the manuscript.

## Conflicts of Interest

The authors declare no conflicts of interest.

## Author Contributions

All authors have made substantial contributions to this manuscript. Dr. Abhishek Vadher was involved in patient care, data collection, manuscript drafting, and literature review. Dr. Mehwish Zeb, Shayne Joseph, Dr. Sujata Kambhatla, and Dr. Chadi Saad contributed to clinical management, critical revisions, and intellectual content.

## Funding

No external funding was received for this study.

## Data Availability

The data that support the findings of this study are available from the corresponding author upon reasonable request.
